# A Case of Systemic Lupus Erythematosus (SLE)-Induced Immune Thrombocytopenia Presented With a Subdural Hematoma

**DOI:** 10.7759/cureus.49958

**Published:** 2023-12-05

**Authors:** Diya M Asad, Salem M Tos, Omar R Khalil, Ahmed H Khammash, Abeer A Awesat, Ahmed M Barbarawi, Mohammad A Isa Assadi, Jawad K Alzaatreh, Majd Mohsen, Alaa Qasem

**Affiliations:** 1 Faculty of Medicine, Al-Quds University, Jerusalem, PSE; 2 Neurosurgery, Al-Quds University, Jerusalem, PSE; 3 Internal Medicine, Al-Quds University, Jerusalem, PSE

**Keywords:** systemic lupus erythematosus, childhood-onset sle, autoimmune disease, subdural hematoma, immune thrombocytopenia

## Abstract

Systemic lupus erythematosus (SLE) is an autoimmune disease that can cause various health problems, including issues with the blood. One common blood-related symptom in SLE is immune thrombocytopenia (ITP), which leads to low platelet counts. In some cases, SLE patients with ITP may develop a rare but serious complication called subdural hematoma (SDH), which is a type of bleeding in the brain. This combination of conditions can be challenging to manage and has a high mortality rate. In a specific case, a 14-year-old girl with chronic ITP developed a sudden headache and was diagnosed with childhood-onset SLE, leading to the development of SDH. The treatment plan had to be adjusted, and a splenectomy was considered. It's important to be aware of the association between SLE, ITP, and SDH, especially in pediatric patients, and to conduct appropriate investigations in cases of severe headaches, to rule out life-threatening causes.

## Introduction

Systemic lupus erythematosus (SLE) is a connective tissue disease that may have multisystem involvement, including the blood [1]. Childhood-onset SLE (c-SLE) patients have more severe manifestations and organ damage than adulthood-onset SLE [2]. One of the frequent hematological manifestations of SLE is immune thrombocytopenia (ITP), which is an autoantibody generated by autoreactive B-cell proliferation and differentiation and abnormal T-cell response against platelets that leads to peripheral platelet destruction, and the platelet count is typically less than 100 × 109/L. The approximate incidence of ITP ranges between 10% to 40% of SLE patients [2,3]. The ITP etiology and the exact mechanism of the underlying immune-mediated thrombocytopenia in SLE remain not fully understood [2]. A rare form of bleeding in ITP is subdural hematoma (SDH), which is defined as a collection of blood between the dura mater and the arachnoid mater. The prevalence of SDH in SLE patients is rare; however, it has a high mortality rate and is difficult to manage [4].

In this article, we report a pediatric patient, presenting with chronic refractory ITP in which SDH occurred, and further investigation was done, which showed that SLE causes the refractory ITP.

## Case presentation

A 14-year-old female patient who is a known case of chronic ITP was referred to our hospital for further evaluation and management of her condition. She presented with a sudden, persistent headache of a one-day duration that was unresponsive to analgesic drugs. She was born to non-consanguineous parents. There were no significant medical or family history findings related to other bleeding disorders. Through additional interrogation, the patient reported the presence of recurrent painless oral ulcers, hair loss, and arthralgia for several months. There were no constitutional symptoms, skin abnormalities, or gastrointestinal or respiratory complaints reported, and no similar medical complaints were reported within the patient's family.

Physical examination showed a conscious, normotensive, and pale patient with normal facial features, palate, and ear position, and no signs of meningeal syndrome or focalization were found. Pupils were equal and reactive bilaterally and spleen enlargement was noted on abdominal examination, the rest of the examination was normal. Upon presentation, her vital signs, Glasgow coma scale (GCS), and platelet count are reported in Table 1.

**Table 1:** Our patient’s vital signs, GCS, and PLT count at presentation GCS: Glasgow coma scale; PLT: platelet

Test	Results	normal range
blood pressure	125/75 mmHg	(90/60-120/80) mmHg
heart rate	110 beats/min	60-100 beats/min
body temperature	37.8°C	(36.11-37.2) °C
Glasgow Coma Score	15/15	
platelet count	8 x 10^9 /L	150-400 x 10^9/L

Additional laboratory investigations were ordered, and the results are documented in Table 2.

**Table 2:** Our patient’s full lab results

Test	Results	normal range
WBC	12K cells/mm^3^, 72% neutrophils, 15% lymphocytes	4,500-11,000/mm^3^, 54-62%, 25-33%
hemoglobin	8 g/dl	13.5-17.5 g/dL (2.09-2.71 mmol/L)
platelets	30 x 10^9 /L	150-400 x 10^9/L
erythrocyte sedimentation rate (ESR)	5 mm/h	Less than age/2 mm/hour
complement C3	0.5 g/L	0.75 to 1.75 (g/L)
complement C4	0.08 g/L	0.16 to 0.48 (g/L)
serum anti-nuclear antibodies	positive (1:3000)	
anti-double-stranded DNA (dsDNA)	positive	
anti-Sm	negative	
anti-SS-A	negative	
anti-Sm/nRNP	negative	
anti-chromatin	negative	
anti-RNP 68	negative	
anti-RNP A antibodies	negative	
antiphospholipid antibody	negative	
lupus anticoagulant	negative	
antiplatelet antibody	negative	
Coombs test	negative	
activated partial thromboplastin time	22 seconds	20-40 seconds
prothrombin time	14.2 seconds	11-14 seconds

Biochemical and hematologic tests, including tumor markers, electrolyte levels, and liver and renal function, demonstrated no abnormalities. Urinalysis yielded normal findings. Chest X-ray and electrocardiography were normal.

Based on the clinical presentation and laboratory results, a diagnosis of childhood-onset SLE with ITP was established. This explains the severity of the disease and its resistance to treatment. The planned intervention of splenectomy was aborted and instead, the patient underwent treatment following the established protocol for SLE.

A non-contrast (CT) scan of the brain was performed, revealing the existence of a left-sided subdural hematoma (SDH).

Emergency treatment was promptly initiated, involving platelet transfusion, intravenous immunoglobulin (IVIG) administration, and two pulses of methylprednisolone (mPSL). Subsequently, a burr hole procedure was performed, followed by a dural incision and evacuation of the hematoma. Considering the presence of severe thrombocytopenia, which was further complicated by the subdural hematoma (SDH) and exhibition of resistance to steroids and IVIG therapy, the treatment plan was adjusted to include a splenectomy.

## Discussion

ITP is the most frequent cause of severe thrombocytopenia in SLE, and it may also include autoimmune hemolysis, which together are referred to as Evans syndrome. ITP typically manifests before the onset of SLE or acutely during flare-ups [5]. According to estimates, 3-15% of ITP patients go on to develop SLE [6]. The symptoms that the patient has described are alopecia, symmetrical bilateral morning joint pain that improves with activity during the day, and painless mouth ulcers. These symptoms may be slight, wax and wane, but they are very common and frequently ignored by treating doctors [7,8].

Childhood-onset SLE (cSLE) is not easy to diagnose; usually, the patient presents with constitutional symptoms (fever, lethargy, weight loss) and the involvement of main organs within three years of disease onset, although some patients present with serious, catastrophic symptoms on arrival to the clinic [9]. When SLE first manifests in children, some traditional correlations, such as Raynaud's phenomenon, Sicca syndrome, and pleuritis, are less frequent, and they manifest more commonly in adults [10].

Even for the most experienced doctors, lupus is the "disease of a thousand faces" and may be challenging to diagnose. SLE symptoms may not meet all SLE criteria at the time of presentation, and since there is no official definition of probable SLE, it is one of the illnesses that should be taken into account in any atypical diagnosis or frequent visits, especially in youngsters.

While SLE involves the nervous system in 75% of cases, symptoms include altered mental status, seizures, cranial nerve disorders, peripheral neuropathy, and paralysis, and an intense headache, as seen in our patient, is seldom reported, especially in the pediatric population [8,11]. Although there are only a few reported cases of subdural hematoma in SLE patients, to the best of our knowledge, this is the second instance of subdural bleeding being reported as a presenting symptom of SLE, particularly in this age group. The management of cSLE has undergone significant modification over time, and the physical and mental adverse effects of corticosteroids necessitate minimizing the dose. As opposed to using steroids or a very low dose of prednisone, hydroxychloroquine is currently used for all patients with SLE without contraindication; higher doses are still used for moderate and severe forms of the condition [12]. After receiving proper care, the prognosis for SLE is typically good. This depends on the severity of the disease, the patient's response to treatment, the occurrence of side effects, and the patient's age, as early disease onset in pediatrics is thought to have a higher morbidity than adult-onset SLE [13], and SLE is thought to be the fifteenth leading cause of morbidity in females between the ages of 10 and 14 in the United States [14].

Based on our experience and other reported cases, we recommend repeated radiological investigations in the form of head CT scans or nuclear magnetic resonance (NMR) imaging to effectively rule out a potentially fatal, but treatable, cause of severe headaches in SLE, especially in the pediatric population [15].

## Conclusions

Systemic lupus erythematosus (SLE) and immune thrombocytopenia (ITP) can have significant implications for patients, including the rare but serious complication of subdural hematoma (SDH). The case of a 14-year-old female with chronic ITP developing childhood-onset SLE and SDH underscores the importance of early recognition and appropriate management to prevent adverse outcomes. Increased awareness among healthcare professionals is vital for timely interventions and improved prognosis, particularly in pediatric patients. Continued research and vigilance are necessary to understand and address the complexities of SLE, ITP, and their associated complications.
